# New plasma diagnostic markers for colorectal cancer: transporter fragments of glutamate tRNA origin

**DOI:** 10.7150/jca.92102

**Published:** 2024-01-12

**Authors:** Changda Ye, Fu Cheng, Luji Huang, Kang Wang, Lin Zhong, Yan Lu, Manzhao Ouyang

**Affiliations:** 1 Department of Gastrointestinal Surgery, Shunde Hospital, Southern Medical University (The First People's Hospital of Shunde Foshan), Shunde, Foshan, Guangdong Province, 528300, China.; 2 The Second School of Clinical Medicine, Southern Medical University, Guangzhou, Guangdong Province, 510080, China.; 3 GCP Center, Shunde Hospital, Southern Medical University (The First People's Hospital of Shunde Foshan), Foshan, 528300, Guangdong, China.

**Keywords:** colorectal cancer, tRNA-derived fragments, i-tRF, glutamate metabolism, liquid biopsy

## Abstract

Colorectal cancer (CRC) is the second leading cause of cancer-related deaths worldwide. Early diagnosis of the disease can greatly improve the clinical prognosis for patients with CRC. Unfortunately, there are no current simple and effective early diagnostic markers available. The transfer RNA (tRNA)-derived RNA fragments (tRFs) are a class of small non-coding RNAs (sncRNAs), which have been shown to play an important role in the development and prognosis of CRC. However, only a few studies on tRFs as early diagnostic markers in CRC have been conducted. In this study, previously ignored tRFs expression data were extracted from six paired small RNA sequencing data in the Sequence Read Archive (SRA) database using MINTmap. Three i-tRFs, derived from the tRNA that transports glutamate (i-tRF-Glu), were identified and used to construct a random forest diagnostic model. The model performance was evaluated using the receiver operating characteristic (ROC) curve and precision-recall (PR) curve. The area under the curves (AUC) for the ROC and PR was 0.941 and 0.944, respectively. We further verified the differences in expression of the these i-tRF-Glu in the tissue and plasma of both CRC patients and healthy subjects using quantitative real-time PCR (qRT-PCR). We found that the ROC-AUC of the three was greater than traditional plasma tumor markers such as CEA and CA199. Our bioinformatics analysis suggested that the these i-tRF-Glu are associated with cancer development and glutamate (Glu)-glutamine (Gln) metabolism. Overall, our study uncovered these i-tRF-Glu that have early diagnostic significance and therapeutic potential for CRC, this warrants further investigation into the diagnostic and therapeutic potential of these i-tRF-Glu in CRC.

## Introduction

Colorectal cancer (CRC) is the second leading cause of cancer-related deaths worldwide, with an expected increase to between 2 and 5 million cases by 2035 1. According to statistics, more than 945,000 people are diagnosed with CRC annually, and about 492,000 people die from CRC 2. With advancements in health awareness and therapeutics, the prognosis of CRC has been improved. It has been shown that the 5-year survival rate of patients with early-stage localized CRC can reach 90%, while that of advanced CRC patients with distant metastases is still less than 15% 3. Thus, increasing the early diagnosis rate is a more effective and socially beneficial option to improve the prognosis of CRC patients.

Current early screening modalities for CRC include colonoscopy, fecal occult blood test (OB), and capsule endoscopy. Colonoscopy is currently the most commonly used tool for CRC screening, its low detection rate, invasiveness, and high cost for screening high-risk CRC groups make it less suitable for meeting patient needs 4. In contrast, simple noninvasive tests like liquid biopsy better meet patients' needs. The carcinoembryonic antigen (CEA) is the only blood biomarker recommended by current guidelines for postoperative surveillance of CRC. However, CEA is not sensitive enough to detect tumor recurrence, and many common factors, such as smoking, infection, inflammatory bowel disease, and liver disease, reduce its diagnostic specificity. Hence, there is an urgent need to discover new noninvasive, inexpensive, and sensitive markers for the early screening and diagnosis of CRC.

The transfer RNA (tRNA)-derived RNA fragments (tRFs) are a class of small non-coding RNAs (sncRNAs) that were previously considered to be non-functional random degradation products of tRNAs. Some tRFs were even previously mistaken as novel microRNAs, such as miR-3676 (ts-3676) 5 and miR-4521 (ts-4521) 6. Nowadays, tRFs are closely associated with numerous pathophysiological processes, and tRFs can be classified based on the site of origin into the following five categories: 5'-half, 3'-half, i-tRF, 5'-tRF, and 3'-tRF. In these classifications, i-tRFs are derived from the internal body of mature tRNAs, including anticodon loops as well as fragments of the D- and T-loops, rather than the 5' and 3' ends 7, and the details of the ribonuclease processing required for their production remain unknown. Moreover, i‐tRFs are highly abundant and may vary depending on gender, population, race, amino acid characteristics, anticodons, tissues, diseases, and disease subtypes. Recent studies have shown that tRFs are associated with the development of various cancer types. A study by Mo et al. found that 5'-tiRNA-Val could regulate the proliferation, migration, and invasion of breast cancer cells through the Wnt/β-Catenin signaling pathway 8. Meanwhile, tRF3008A has been shown to inhibit the metastasis and progression of CRC by destabilizing FOXK1 in an AGO-dependent manner 9. Furthermore, 5'-tRF-Gly in the plasma of CRC patients was identified as an early diagnostic marker for CRC 10. Although tRFs are currently being studied in the context of various tumors, few studies have been done in relation to CRC, especially on their potential role as an early diagnostic biomarker.

During tumor development, the metabolic reprogramming of amino acids, as one of the three major nutrients in the body, plays a significant role. For example, tumor cells can promote their growth and suppress T-cell activity through metabolic reprogramming and the competitive uptake of glutamine, leading to immunosuppression 11,12. Moreover, amino acid metabolism was found to be strongly associated with CRC. Peng et al. found that amino acid metabolism-related genes were associated with the immune microenvironment in CRC patients, and could be used as biomarkers to predict patient prognosis and immunotherapeutic response 13. In addition, key enzymes of amino acid metabolism in tumor-associated macrophages (TAMs) have been suggested to be involved in the CRC immune escape process by affecting programmed cell death (PCD) and polarization of TAMs 14. Thus, tRFs involved in amino acid metabolism might be affected by metabolic reprogramming, leading to changes in their intracellular content, and could further influence the course of CRC.

Interestingly, we found several studies which performed small RNA sequencing (RNA-seq) analysis by deleting tRFs as confounding signals, resulting in the loss of many valuable tRFs sequencing data 15. In line with this, we obtained six small RNA-seq data from the SRA database to explore and identify tRFs with significant value. After data collation, random forest algorithm screening and real-time quantitative PCR (qRT-PCR) validation, we finally identified three tRFs associated with Glu (tRF-22-RNLNK88KL (tRF-22), tRF-27-Z3M8ZLSSXUL (tRF-27) and tRF-32-0668K87SERM4P (tRF-32)), which were significantly highly expressed in tissues and plasma of CRC patients and had the ability to perform early diagnosis of CRC. In addition, bioinformatics analysis suggested a correlation between tRF-22/27/32 and cancer development and glutamate-glutamine (Glu-Gln) metabolism, among others. It is noteworthy that in tumor cells the metabolism of Gln, which is the most abundant amino acid and a major cellular energy substrate besides glucose, is significantly increased 16. Overall, our results indicate that tRF-22/27/32 are potential early diagnostic markers and therapeutic targets for CRC and deserve further study and exploration.

## Method and materials

### Datasets

The small RNA-seq datasets (SRP107326, SRP166942, SRP183064, SRP193100, SRP344867, and SRP289772) used in the training and independent validation sets were obtained from the SRA public repository. Meanwhile, six datasets of clinical information and correlation heat maps of the required mRNA expression matrix (GSE121842) 17 were obtained from the Gene Expression Omnibus (GEO) database. Moreover, the RNA-seq data required for Gene Set Enrichment Analysis (GSEA) were obtained from The Cancer Genome Atlas (TCGA) database. All sequencing files were processed through format conversion, removal of adapters, low quality filtering, and final matching using MINTmap 18 to obtain the tRFs expression levels (Counts and RPM, from MINTmap output files), which were processed to obtain the tRFs expression matrix. The missing values in the expression matrix were filled with MetImp 1.2 19.

### Statistical analysis

Differential analysis was performed using the limma package (RPM matrix, version 3.52.4) 20 and the DEseq2 package (Counts matrix, version 1.36.0) 21, and the results of the differential analysis were taken as the intersection. We identified 317 tRFs from the intersection of the difference analysis results, and then we used the randomForest package (version 4.7.1.1) 22 to construct a diagnostic model of CRC for the top 100 tRFs. We then selected three i-tRF-Glu (tRF-22-RNLNK88KL, tRF-27-Z3M8ZLSSXUL, tRF-32-0668K87SERM4P) from the top 30 of the Gini index, which were again used to construct the diagnostic model. The model performance was evaluated using ROC and PR curves and was tested using an independent validation set (SRP289772). All the above analyses were performed in the R programming language (version 4.2.1). Meanwhile, the logistic regression analysis was performed using IBM SPSS Statistics (version 18), and ROC curves were plotted. The results of the tissue and plasma gene expression analyses were analyzed using GraphPad Prism (version 8.0.2) for paired and unpaired t-tests. The clinical data were processed using IBM SPSS Statistics (version 18). The correlation of all available clinically dichotomous data with the high/low expression levels of tRF-22/27/32 was tested using Pearson's chi-squared test or Fisher's exact test. *P<0.05, **P<0.01, ***P<0.001, ****P<0.0001, ns indicates not significant. A *P*-value of <0.05 was considered statistically significant.

### Target prediction and enrichment analysis

We used miRDB 23, TargetScan 24, TargetRank 25, and RNAhybrid^26^ to predict the target genes of tRF-22/27/32. The intersection of the four databases was used to select out the target genes that were predicted with high scores. The target genes were then analyzed by the Kyoto Encyclopedia of Genes and Genomes (KEGG) and Gene Ontology (GO) using the Database for Annotation, Visualization, and Integrated Discovery (DAVID) 27,28, and the analysis results were downloaded and visualized using the R package ggplot2 (version 3.4.0). GSEA was performed using the R package ClusterProfiler (version 4.4.4) 29. The network diagram was visualized using Cytoscape software (version 3.9.1).

### Patient tissue samples

Human specimens were collected and used in this study with the approval of the ethics committee of Shunde Hospital, Southern Medical University (The First People's Hospital of Shunde Foshan), Shunde, Foshan, Guangdong Provinc, China. The malignant tissues were collected from patients diagnosed with CRC by tumor histopathological analysis and underwent colorectal resection in Shunde Hospital, Southern Medical University (The First People's Hospital of Shunde Foshan) from 2021 to 2023. All specimens were collected with the informed consent of the patients, and all patients had not received radiotherapy or chemotherapy before surgery. A total of 24 pairs of CRC and normal paracancerous tissues were used to verify the expression of tRF-22/27/32. Subsequently, we collected blood samples from 40 CRC patients and 40 normal volunteers to test the expression levels of tRFs in plasma. The study was conducted in accordance with the Declaration of Helsinki. Detailed inclusion and exclusion criteria for patients enrolled in this study were as follows.Inclusion criteria included. (1) histologically confirmed colon or rectal cancer, (2) no other malignancies in combination, (3) Chinese male or female subjects aged ≥18 years, and (4) voluntary signing of informed consent. Exclusion criteria: (1) Previous receipt of first-line systemic anti-tumour therapy for metastatic CRC (including systemic chemotherapy, molecular targeted drug therapy, biotherapy and other investigational therapeutic agents); (2) Treatment with other concurrent anti-tumour therapy, long-term systemic immunotherapy; (3) History of malignancy other than colorectal cancer within the last 5 years; (4) Participation in a clinical trial of another drug within 30 days prior to screening; (5) History of autoimmune disease or other medical conditions; (6) Other conditions that, in the opinion of the investigator, preclude enrolment of subjects.

### Gene expression analysis by qRT-PCR

Total RNA was extracted from CRC tissue and plasma using RNAiso Plus (Takara, Japan). Reverse transcription was performed with specific tRFs stem-loop RT primers, and cDNA amplification was performed by qRT-PCR using the ChamQ Universal SYBR qPCR Master Mix reagent (Vazyme, China) and the QuantStudio™ 5 Real-Time Fluorescence PCR System (Thermo Fisher, USA). U6 was used as an internal control for tRFs, and we used the -ΔCt method to detect the expression of genes related to the internal control. Each assay was repeated three times. The agarose gel electrophoresis was used to check the uniqueness of the qRT-PCR products. To further confirm the sequence of the qRT-PCR products, we performed Sanger sequencing experiments. The primer sequences are summarized in **Table S1**.

## Results

### Identification of differentially expressed tRFs in CRC

**Figure 1:**
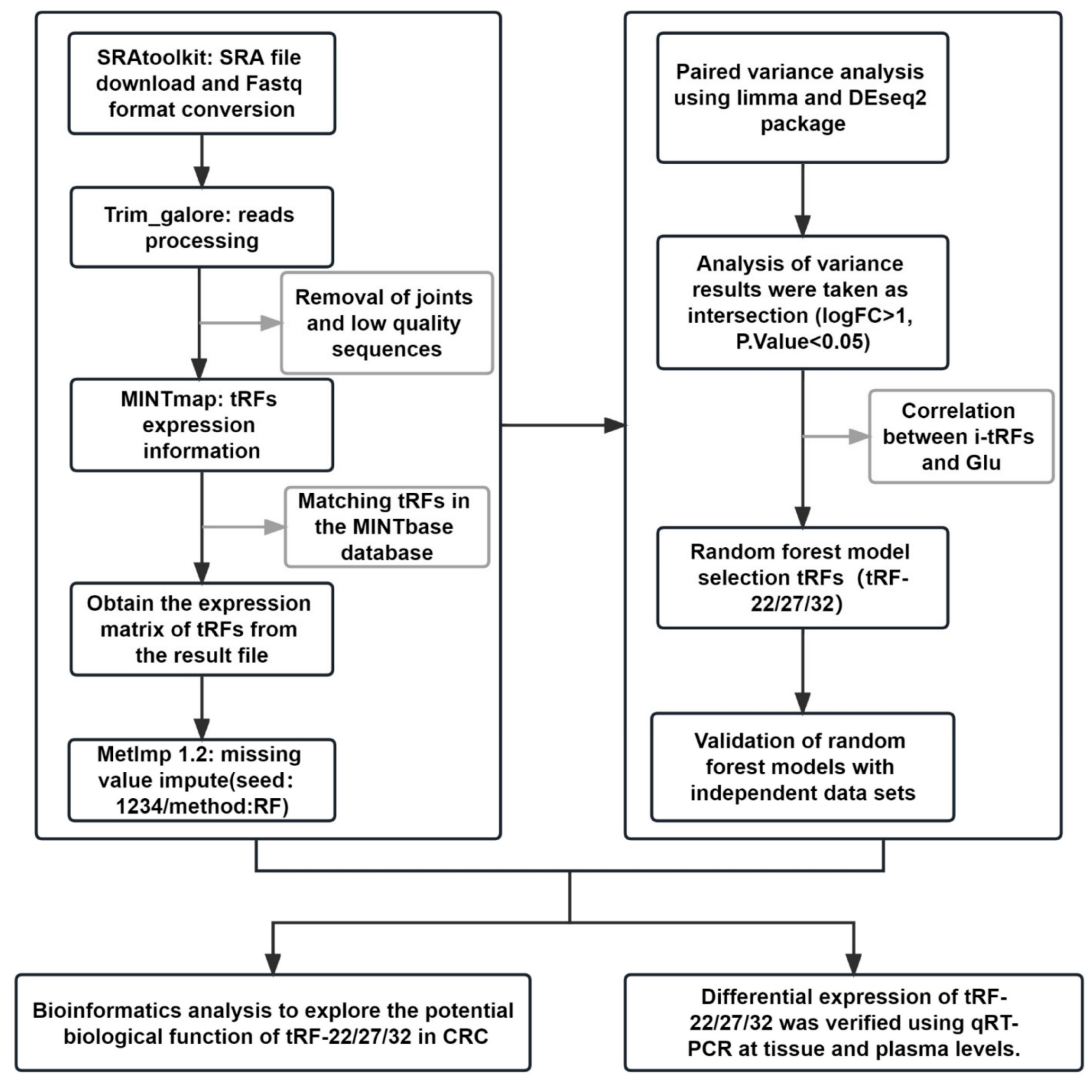
** Workflow of data process.** Expression levels of tRFs were extracted using small RNA sequencing data from the SRA database of paired colon cancer and paracancerous tissues (Upper left panel). Random forest analysis was used to screen tRFs, and 3 tRFs were finally identified (upper right panel). Bioinformatics analysis: target gene prediction, function and pathway enrichment analysis (lower left panel). Expression of the 3 tRFs was verified at tissue and plasma levels using qRT-PCR (lower right panel).

**Figure 2:**
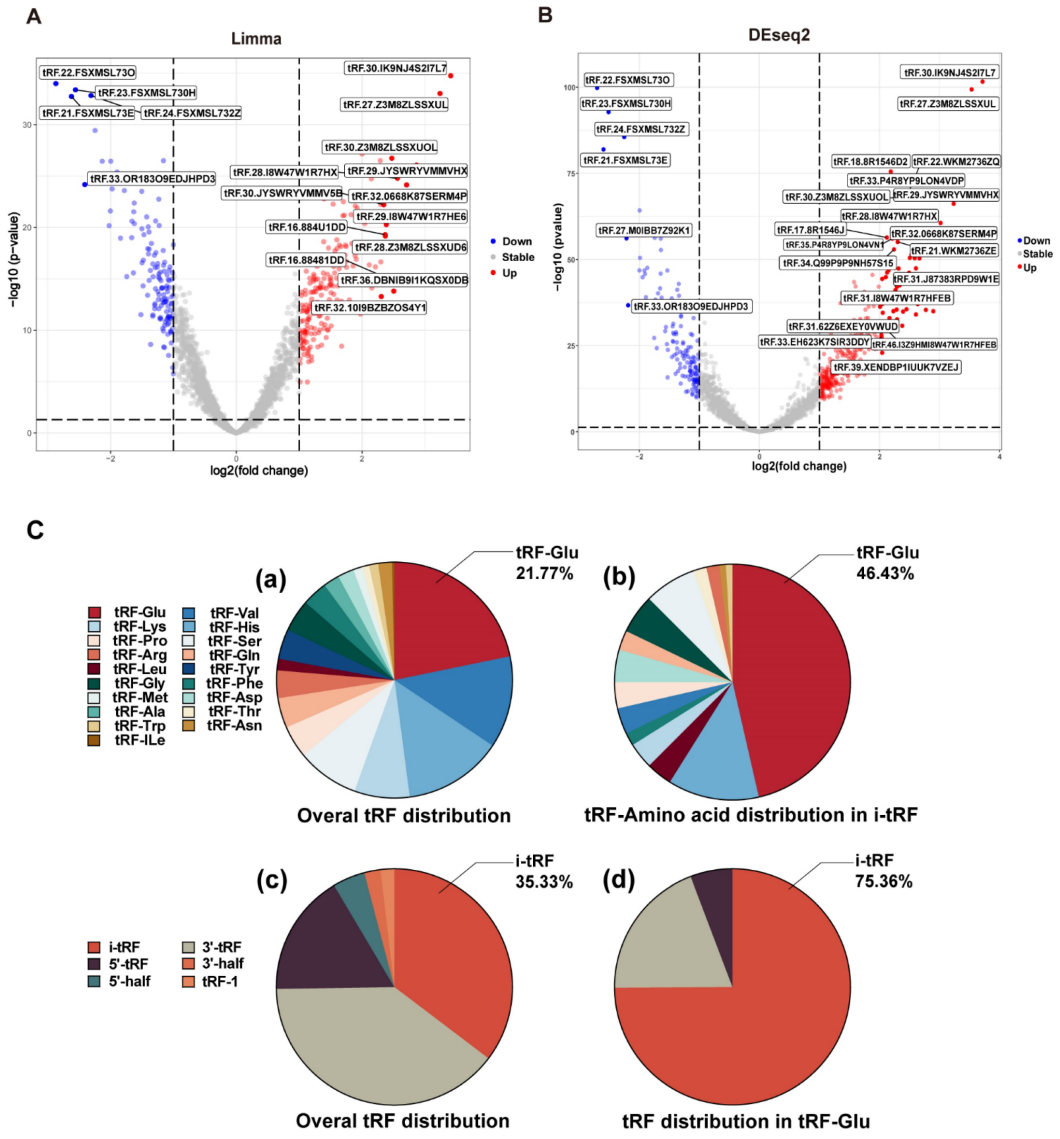
** Differential analysis and the composition analysis of the intersection tRFs.** (A) Volcano plot of Limma package differential analysis. (B) Volcano plot of Deseq2 package differential analysis. (C) Composition analysis of the intersection tRFs: (a) tRF-Glu accounted for 21.77% of the intersection tRFs. (b) tRF-Glu accounted for 46.43% of the i-tRFs. (c) i-tRF accounted for 35.33% of the intersection tRFs. (d) i-tRF was 75.36% in tRF-Glu.

**Figure 3:**
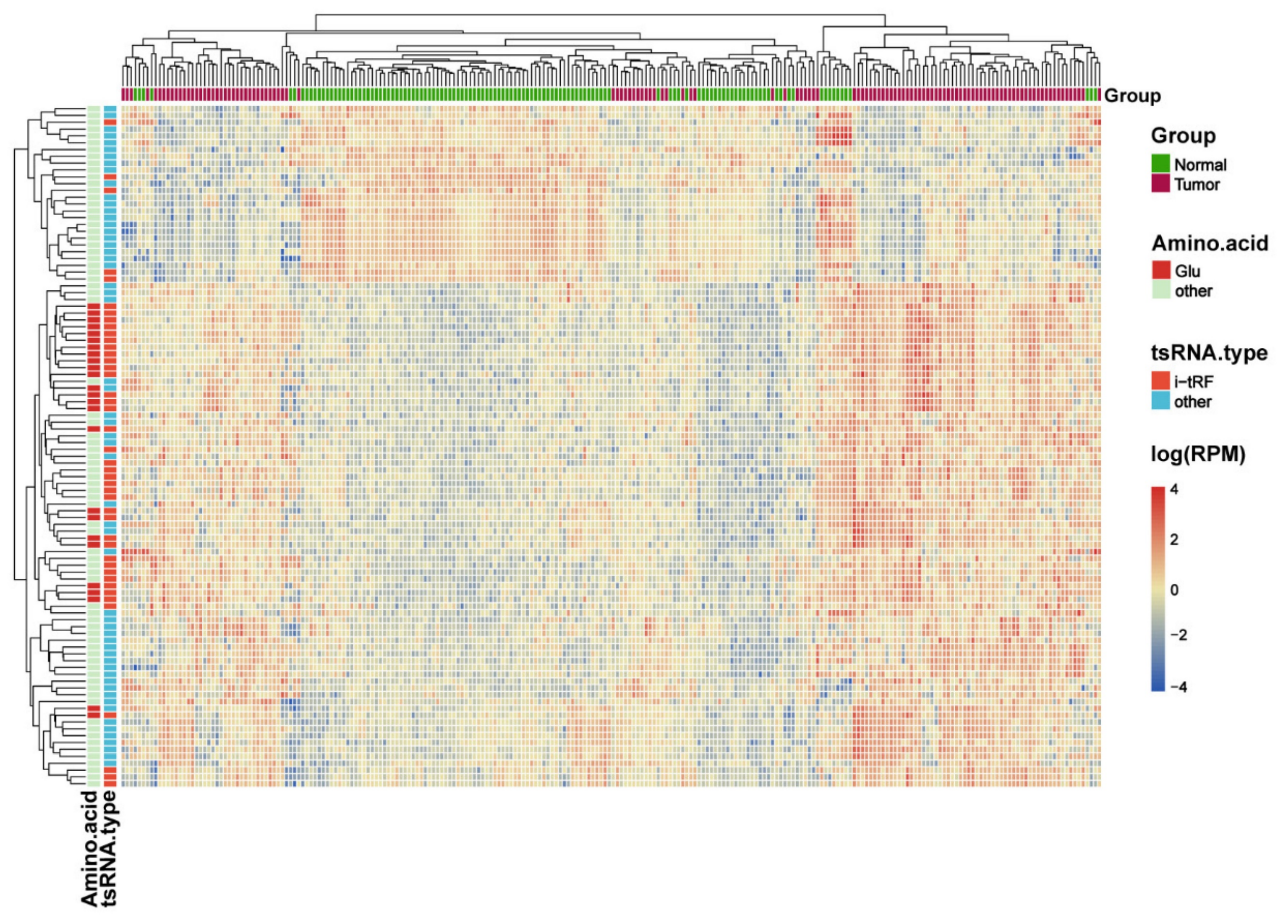
** Heat map of the expression of the top 100 difference tRFs.** Differential analysis of the tRFs using Limma package and DEseq2 package, the expression heat map displays the top 100 intersecting tRFs of LogFC absolute values.

The whole data processing workflow performed in this study is illustrated in** Figure 1**. Moreover, the clinical information from the 6 datasets we used is summarized in **Table 1**. Following the extraction of the tRFs expression matrices from the SRA files for 240 pairs of cancer and paracancerous CRC tissues, we performed paired differential analysis using the limma **(Figure 2A)** and DEseq2 packages **(Figure 2B)**, respectively (The top 50tRFs in the intersection of differential analysis results are displayed in **Table S2**). The intersection of the two analyses was used to obtain 317 differentially expressed tRFs (***P***<0.05, |logFC|>1; **Figure S1**). Upon collating the information on the intersecting differential tRFs **(Table S2)**, we observed that there seems to be a close correlation between i-tRFs and Glu-tRNA-derived tRFs (tRF-Glu; **Figure 2C**). The tRF-Glu ranked first with 21.77% of the intersecting differential tRFs, and tRF-Glu also ranked first with 46.43% of the i-tRFs. Meanwhile, i-tRFs ranked second with 35.33% of the intersecting differential tRFs, while i-tRFs ranked first with 75.36% of the tRF-Glu. Furthermore, we ranked the expression of the top 100 intersection tRFs in descending order of the absolute value of logFC from the heat map** (Figure 3)**. In the row clustering, we also observed that there is a large correspondence between i-tRFs and tRF-Glu. The chi-square test for i-tRFs and tRF-Glu showed a χ^2^pearson=61.858, and a *P*-value<0.001, indicating a significant correlation between the two.

### Selecting out three i-tRF-Glu to construct CRC diagnostic models

**Figure 4:**
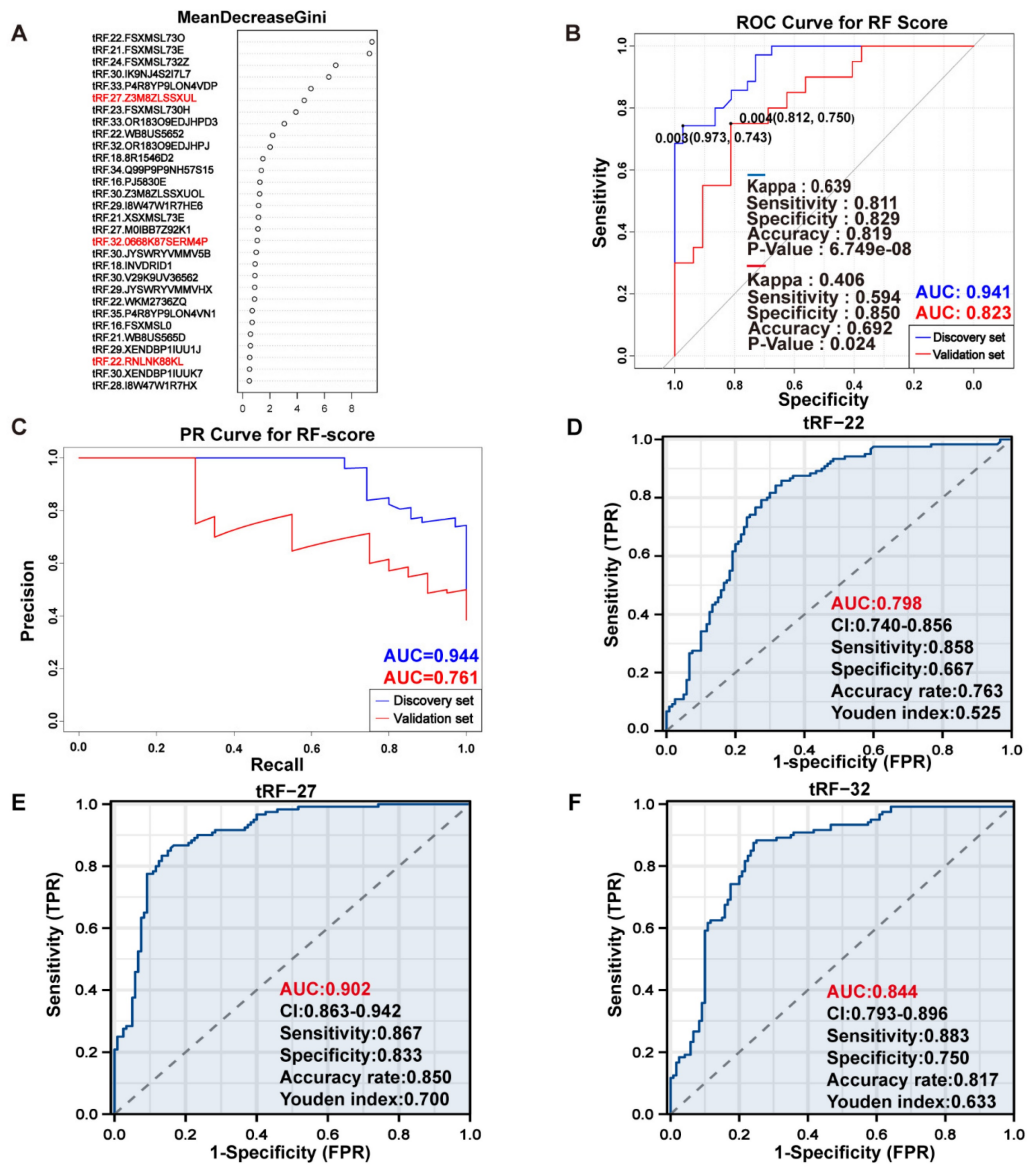
** Exploring the diagnostic efficacy of tRF-22/27/32 for CRC.** (A) Random forest RF mean decrease Gini score rank. (B) ROC curves of the random forest diagnostic model in the training set and independent validation set. (C) PR curves of the random forest diagnostic model. (D~F) Separate ROC curves for tRF-22/27/32 in the training set (logistic regression analysis).

The intersection of the differential analysis results was explored to identify 317 tRFs (***P***<0.05, |log2 FC|>1), which were then subsequently used to construct a random forest model using the RandomForest package. Combined with the analysis of the composition of the differential tRFs as a result of the differential analysis, we concluded that i-tRF-Glu may play an important role in the development of CRC, and therefore we screened for i-tRF-Glu in the top 30 tRFs. Finally, three i-tRF-Glu with high expression in colorectal tissues were selected: tRF-22-RNLNK88KL (tRF-22), tRF-27-Z3M8ZLSSXUL (tRF-27), and tRF-32-0668K87SERM4P (tRF-32), among the top 30 tRFs ranked by Gini index** (Figure 4A)**. These tRFs were then used to reconstruct the random forest diagnostic model again. Model performance was evaluated by testing out-of-bag (OOB) samples. The diagnostic efficacy of the established diagnostic model was evaluated using ROC and PR curves, and the test results showed values of ROC -AUC=0.941 **(Figure 4B)** and PR -AUC=0.944 **(Figure 4C)**. To further test the diagnostic efficacy of the model in the real world, we selected SRP289772 as an independent validation set, which contains sequencing data from 32 normal volunteers and 20 CRC patients. We calculated a diagnostic RF-score for the independent validation dataset (SRP289772) based on the OOB predicted probabilities, the PR-AUC and ROC-AUC of the established model were found to be 0.761 and 0.823 in the independent validation set **(Figure 4B-C)**. We also modeled the tRF-22/27/32 separately using logistic regression and plotted the ROC curves separately with ROC-AUCs of 0.798, 0.902, and 0.844 for each of these tRFs **(Figures 4D-F)**. And then We analyzed their expression differences in cancer and normal using paired t-test and unpaired t-test in the training set and independent validation set, respectively. The results revealed that the three were significantly highly expressed in CRC both in the training and independent validation sets and statistically different (***P***<0.05), except for tRF-22 which maybe not statistically different in the independent validation set due to insufficient sample size.** (Figure S2)**.

### Potential biological function of tRF-22/27/32 in the development of CRC

**Figure 5:**
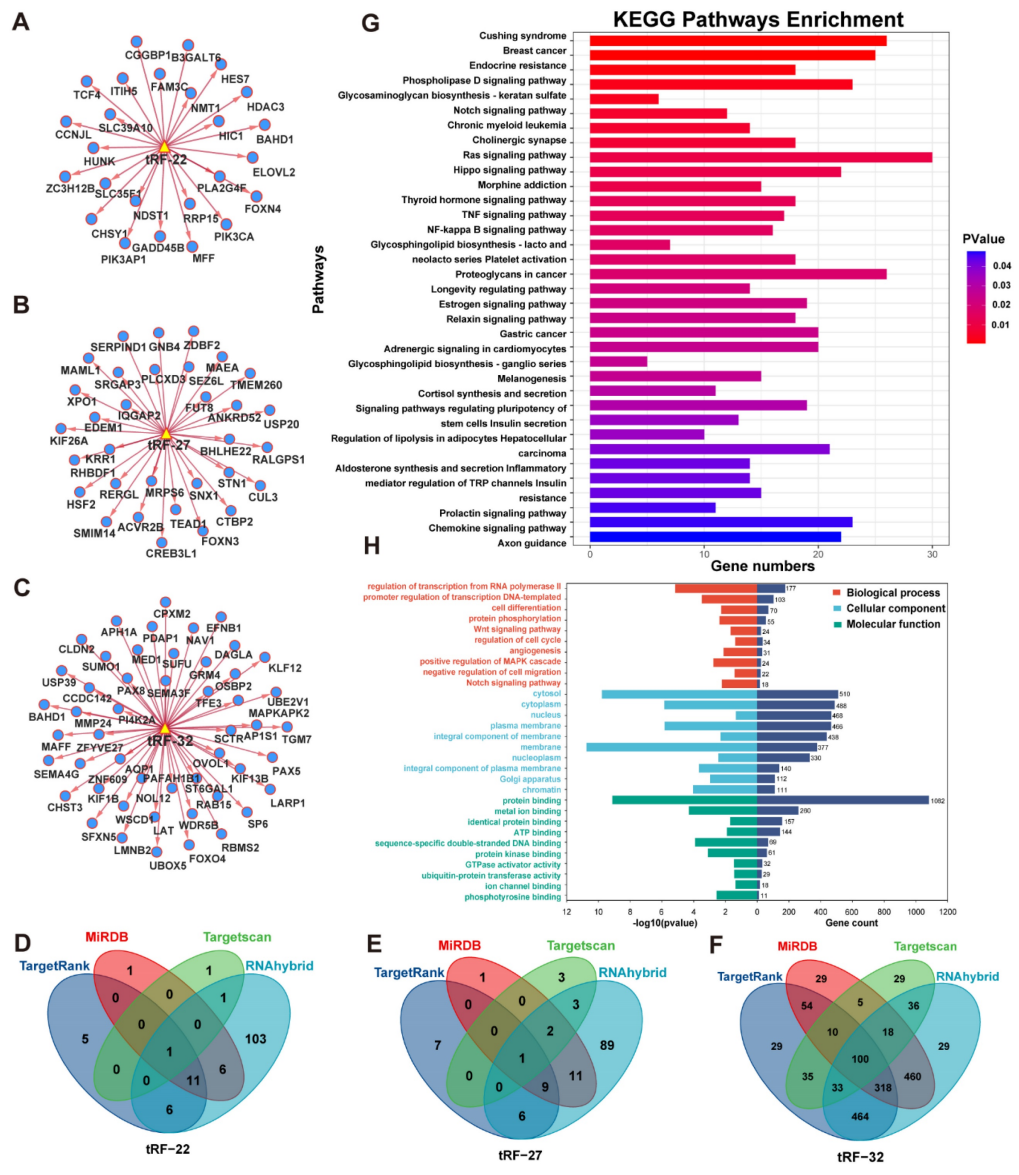
** Enrichment analysis of database predicted target genes (intersection).** (A~C) Network analysis of tRF-22/27/32 with predicted target genes. (D~F) Venn diagram of four database predicted target genes of tRF-22/27/32. (G) KEGG enrichment analysis of tRF-22/27/32 target genes. (H) GO enrichment analysis of tRF-22/27/32 target genes.

The target genes of tRF-22/27/32 were predicted using four databases: TargetRank, TargetScan, RNAhybrid, and miRDB. The tRF-mRNA interaction network was then established using some of the predicted target genes **(Figure 5A-C)**. Subsequently, all target genes that were predicted in two or more databases were extracted yielding a total of 1,583 genes **(Figure 5D-F)**.

The extracted gene list was then subjected to KEGG and GO enrichment analysis in the DAVID database. The enrichment analysis results were visualized using the R programming language **(Figure 5G-H)**. In the KEGG enrichment analysis, most genes were enriched in the RAS signaling pathway. The other genes were found to be involved in numerous signaling pathways which are closely related to tumor development, such as TNF/NF-kappa/Notch/Hippo **(Figure 5G)**. Meanwhile, in the GO enrichment analysis, the terms biological process (BP), fractional cell composition (CC), and molecular function (MF) each displayed 10 pieces of information with *P*-values less than 0.05 **(Figure 5H)**. Among them, the important biological functions are transcriptional regulation of the RNA pol II promoter, cell differentiation, and protein phosphorylation, while most of the target genes were mainly localized in the cytoplasm, nucleus, and cell membrane. The most important molecular function is protein binding.

For further analysis, we obtained the CRC RNA-seq data from TCGA, extracted the gene expression matrix, and performed differential analysis. The results of the differential analysis were intersected with the target genes to obtain the differential expression information of target genes in CRC. Using GSEA, six enriched pathways emerged with *P*.adj<0.05, including the Wnt signaling pathway, G2 M checkpoint, and RNA metabolism, L1CAM interactions, potential therapeutics for Sars, malignant pleural mesothelioma **(Figure S3)**.

### tRF-22/27/32 are involved in the regulation of Glu metabolism

**Figure 6:**
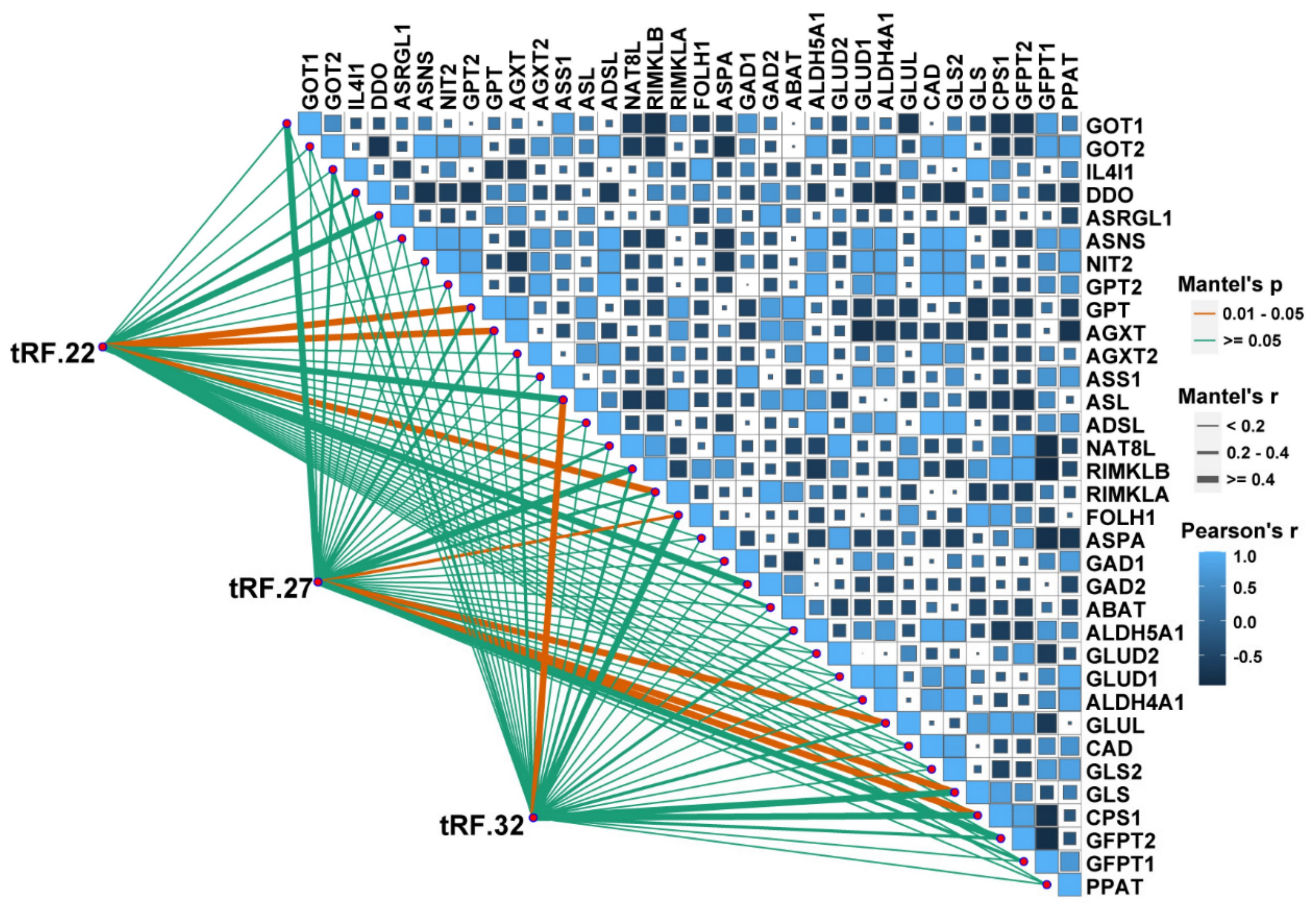
** Correlation analysis of tRF22/27/32 with glutamate metabolism-related genes.** The bottom left corner is the connection is the expression analysis of tRF-22/27/32 with glutamate-related genes, and the top right corner is the expression correlation analysis between glutamate-related genes. Glutamate-related gene expression information was obtained from the GEO database (GSE121842).

A correlation analysis was performed to explore the association between tRF-22/27/32 and Glu metabolism. The RNA-seq data GSE121842 (SRP166942) from the GEO database was used to obtain the mRNA expression matrix **(Figure 6)**. According to Mantel's *P*<0.01, tRF-22 is associated with the expression of GTP, alanine-glyoxylate aminotransferase (AGXT), and ribosomal modification protein RimK-like family member A (RIMKLA). RIMKLA has also been suggested to be involved in the Gln family amino acid metabolism 30. Meanwhile, tRF-27 was significantly correlated with the expression of glutamine ligase (GLUL), GLS, G protein pathway inhibitor 1 (GPS1), and folate hydrolase 1 (FOLH1) genes. Among these, GLUL is mainly involved in catalyzing the synthesis of Gln from Glu and ammonia in an ATP-dependent reaction. This protein plays a role in ammonia and Glu detoxification, acid-base homeostasis, cell signaling, and cell proliferation 31, while GLS catalyzes the hydrolysis of Gln to Glu and ammonia 32. Furthermore, tRF-32 was shown to correlate with the expression of argininosuccinate lyase (ASL), which primarily catalyzes the reversible hydrolysis of argininosuccinate to arginine and fumarate - an important step in the detoxification of ammonia by the liver through the urea cycle 33. In addition, pathway analysis of these genes was performed using the online database DAVID. The results showed that these genes are associated with the anabolism of amino acids such as Glu, alanine, and aspartate and that there is a close relationship between these genes and the development of CRC (Figure S4).

### The tRF-22/27/32 can serve as plasma markers for CRC screening

**Figure 7:**
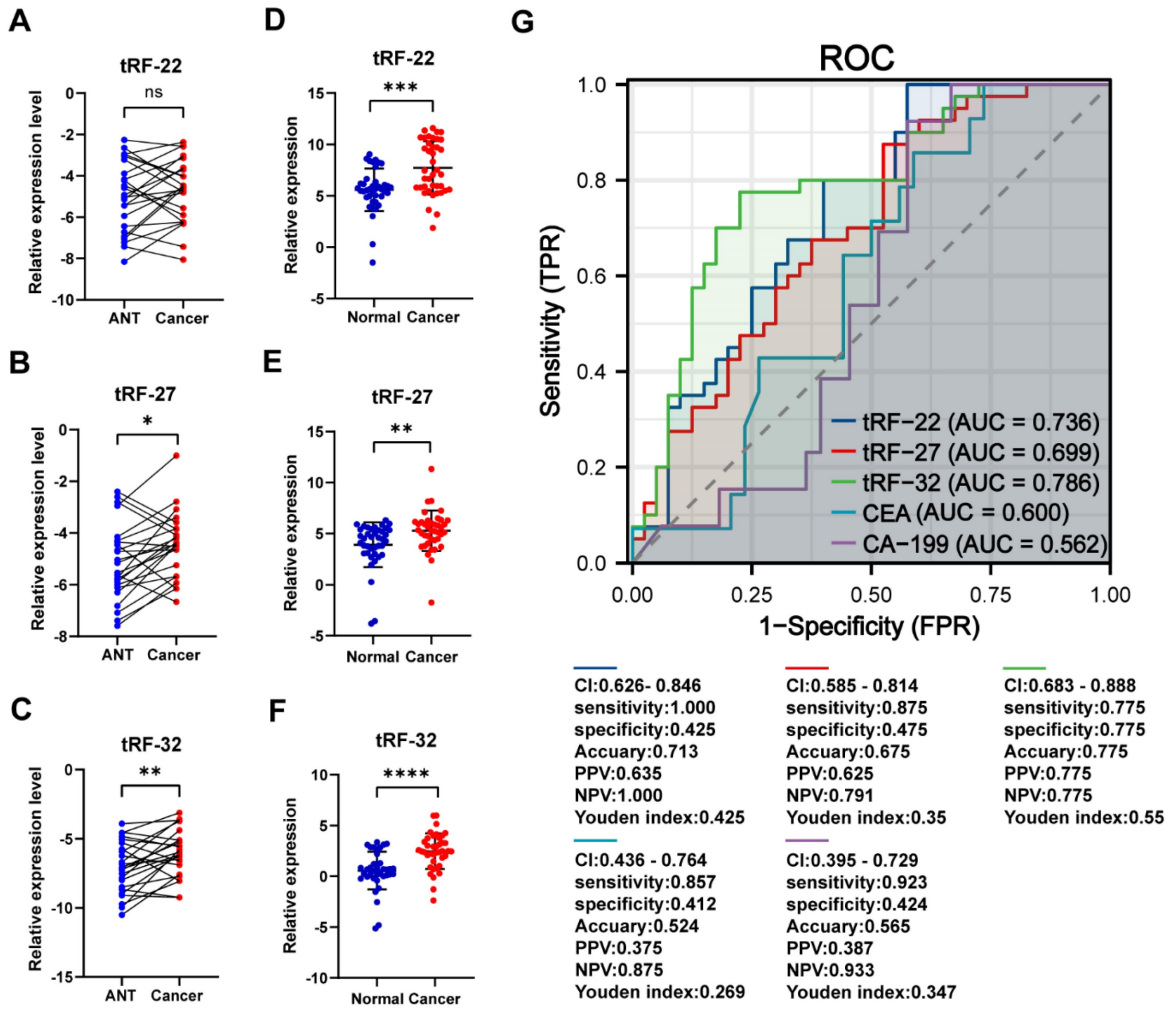
** qRT-PCR to validate the differential expression of tRF-22/27/32.** (A~C) qRT-PCR validation of tRF-22/27/32 expression differences in tissues. (D~F) qRT-PCR validation of tRF-22/27/32 expression in plasma of CRC patients and normal volunteers. (G) ROC curves of tRF-22/27/32, CEA, and CA199 in CRC patients and normal individuals.

We collected cancer and paracancerous tissues from 24 CRC patients for gene expression analysis to further validate the differential expression of tRF-22/27/32. The results showed that both tRF-27/32 had significantly higher expression in cancer tissues (*P*-values=0.012 and 0.006, respectively; **Figure 7A-C**), which is consistent with the results obtained from the small RNA-seq data. Subsequently, we evaluated the CRC diagnostic efficacy of tissue qRT-PCR results for tRF-22/27/32 in the same model as the miRNA sequencing dataset (The results are shown in **Figure S5**). To further demonstrate the correctness of the amplification products of the qRT-PCR of the three tRFs, we performed agarose gel electrophoresis (Result is shown in **Figure S6**) and Sanger sequencing experiments (**Figure S7**) using the products of the three qPCRs, and the results showed that the products of the three qRT-PCRs were specific and sequenced correctly.

**Figure 8:**
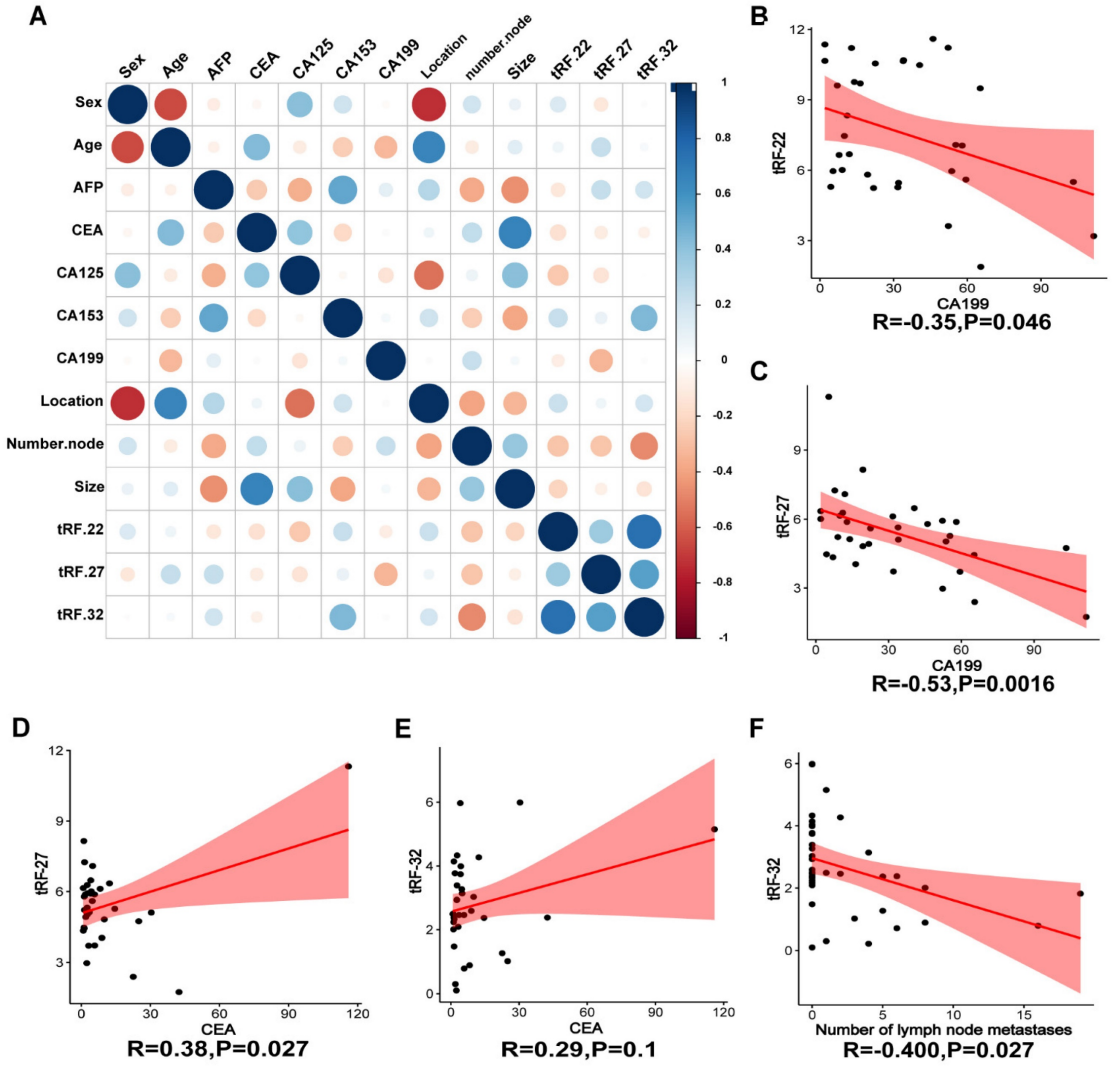
** Correlation analysis of clinical information.** (A) Heat map of correlation between patient clinical information and tRF-22/27/32. (B~C) Correlation analysis of CA199 and tRF-22/27. (D~E) Correlation analysis of CEA and tRF-27/32. (G) Correlation between the number of lymph node metastases and tRF-32 was demonstrated.

To verify the expression of the three tRFs in plasma and determine the possibility of using them as diagnostic markers for CRC, blood samples were collected from 40 CRC patients and 40 normal volunteers. The clinical characteristics of the patients are summarized in **Table 2**. Gene expression analyses showed that all these tRFs were highly expressed in the blood of CRC patients compared with that of normal volunteers (*P-*value=0.025, 0.002, and 0.005, respectively; **Figure 7D-F)**. To further determine the potential of these tRFs as diagnostic markers, the ROC curve for each tRFs was plotted after collecting clinical data from patients. The results showed that tRF-32 had the highest ROC-AUC of 0.786, while tRF-22/27 had 0.736 and 0.699, respectively **(Figure 7G)**. Moreover, all three ROC-AUCs were better than that of traditional tumor markers CEA (0.600) and CA199 (0.562). Subsequently, we analyzed the relationship of the tRFs with clinical data, and found that these tRFs may be associated with CEA levels, the number of lymphatic metastases, and tumor size and location in CRC patients **(Figure 8A)**. Furthermore, the expression level of tRF-32 in CRC tissues differed with age (*P*=0.013), while both lymph node metastasis (*P*=0.011) and vascular invasion (*P*=0.005) of the tumor correlated with the expression of tRF-32. Meanwhile, tRF-27 levels in CRC were significantly higher in stage III than in stage I cancer. However, the indicators of mismatch repair (MMR), which symbolize the tumor mutation load and tumor differentiation 34, showed no correlation with these tRFs **(Table 2)**. Finally, we explored the correlation between tRF-22/27/32 and traditional tumor markers and found a negative correlation between tRF-22 and tRF-27 with CA199 in plasma **(Figure 8B-C)**, and a positive correlation between tRF-27 and CEA **(Figure 8D)**. These results suggest that tRF-22/27/32 can be used as biomarkers for early diagnosis of CRC and may mediate CRC development by affecting tumor invasion.

## Conclusions and Perspectives

CRC has become the third most common cancer in men and the second most common in women. By 2035, the number of CRC cases worldwide is expected to increase to 2-5 million, which will cause a significant socioeconomic burden. Currently, surgical resection, supplemented with chemotherapy and radiotherapy, is the main treatment for CRC 35. Although targeted and immunotherapy have achieved some success in CRC in recent years, increasing the early diagnosis rate seems to be a more effective and socioeconomic option to improve the clinical prognosis of CRC. It has been shown that the 5-year survival rate for advanced CRC patients with distant metastases is less than 15%, whereas the 5-year survival rate for patients with early-stage localized CRC can reach 90% 3. Current screening modalities for CRC include colonoscopy, OB, and tumor marker (e.g., CEA and CA199) tests. However, these methods can also present some challenges and inconveniences, such as the invasive and expensive nature of colonoscopy 36, the poor sensitivity and specificity of the OB 37, and the susceptibility to interference and the long half-life of CEA, which cannot indicate the presence of early-stage tumors 38. In contrast, tRFs, which are highly base-modified, highly enriched in various biological fluids, and have higher levels and stability, are promising sncRNAs that have garnered significant attention in recent years and have the potential to be used as plasma markers for tumor screening. Related studies have shown that tRFs have great potential value in the diagnosis of CRC, for example, chen et al. found that TRF-phe-gAA-031 (AUC=0.755) and tRF-VAL-tca-002 (AUC=0.731) had high diagnostic efficacy in the diagnosis of CRC 39. In addition, Wu et al. found that 5'-tRF-GlyGCC (AUC=0.882) had higher diagnostic efficacy compared to traditional tumour markers (CEA (AUC=0.762), CA199 (AUC=0.557) 10. Therefore exploring tRFs as a diagnostic marker for CRC is a very promising field. However, it is worth noting that most current studies have ignored some of the tRFs as confounding signals when performing small RNA-seq analyses, which has led to the loss of much valuable tRFs data. Therefore, it is very valuable to explore the tRFs data from small RNA-seq studies for CRC.

By integrating multiple small RNA-seq data and mining the neglected tRFs through MINTmap, we found that the differentially expressed tRFs in CRC were mainly composed of i-tRF and tRF-Glu. Previous studies have reported that the expression of tRFs was abnormal in tumors. For example, Huang et al. found that tRF-31-U5YKFN8DYDZDD was highly expressed in gastric cancer 40. Other studies demonstrated high expression of tRF-Leu-CAG in non-small cell lung cancer 41. In contrast, Wu et al. found by sequencing small RNAs in the plasma of CRC patients and normal subjects that the percentage of plasma i-tRF in normal subjects was only 17.57%, while in CRC patients, it reached 25.31%, second only to 5'-tRF (57.22%), indicating the overexpression of i-tRFs during CRC development. These findings are consistent with our study. However, our screening of differential tRFs by integrating multiple small RNA-seq data may lead to more convincing conclusions compared to individual sequencing data. Furthermore, we found that there was a significant correlation between i-tRFs and tRFs-Glu among the differentially expressed tRFs in CRC (*P*<0.001). Similarly, Wu et al. also found that among the 5 '-tRFs with the highest content, tRFs-Glu accounted for 11.46% in CRC patients, which was significantly higher than 6.30% in normal subjects 10, further supporting our hypothesis that i-tRFs participate in the occurrence and development of CRC and may be related to Glu metabolism. tRNAs are are involved in the transport of amino acids during protein synthesis. Interestingly, CRC is usually accompanied by a significantly abnormal amino acid metabolism 42. In a study by Xie et al., they characterized the amino acid metabolism of CRC based on the expression status of 358 amino acid metabolism-related genes and divided CRC into AA1 and AA2 types. The AA1 subtype is characterized by a weak amino acid metabolism activity, a high tumor mutation load, significant immune cell infiltration, and poor prognosis, although it may benefit from irinotecan, anti-PD-1, and CTLA-4 immunotherapy. Meanwhile, the AA2 subtype exhibits strong amino acid metabolic activity, increased sensitivity to 5-fluorouracil and oxaliplatin, and generally has a good prognosis 43. That means the metabolism of abnormal amino acids in CRC not only regulates tumor progression but also affects treatment response and patient prognosis. tRFs are likely to serve as a bridge between the occurrence and development of CRC and amino acid metabolism. Therefore, we selected three i-tRF-Glu (tRF-22/27/32) that were significantly highly expressed in CRC and were later verified in tissue samples. The ROC curves showed that these tRFs could distinguish tumor from non-tumor tissues. At the same time, tRF-32 was associated with lymph node metastasis and nerve/vascular invasion of the tumor. Hence, we hypothesize that tRF-22/27/32 are involved in the occurrence and progression of CRC, as well as in the metabolism of Glu-Gln. These intriguing results urge us to continue to explore the molecular contribution of tRF-22/27/32 in the occurrence and development of CRC.

We further performed enrichment analyses of the target genes of tRF-22/27/32 and found that these genes were mainly enriched in signaling pathways, such as WNT, MAPK, and Notch, and were also involved in biological processes, such as G2M cell cycle checkpoint and L1CAM adhesion molecule interactions. The role of these pathways in CRC is relatively clear. For example, the Wnt signaling pathway is closely related to CRC. It can participate in the initiation and development of CRC by integrating the stem cell characteristics of tumor cells and modulating apoptosis, autophagy, inflammation, immunity, and chemoresistance 44. On the other hand, amino acid metabolism has been shown to also interfere with the MAPK signal pathway. Xue et al. found that branched chain amino acids α Ketate dehydrogenase kinase (BCKDK) directly mediates the phosphorylation of Ser221 of ERK1 and promotes CRC progression by activating the MAPK signaling pathway 45. In addition, a variety of sncRNAs are involved in the regulation of the Notch signaling pathway in CRC. It has been shown that miR-34a 46, miR-195-5p 47 can regulate the levels of Notch1, Notch2 in CRC, respectively, and participate in the induction of CRC cell apoptosis and inhibition of CRC proliferation, migration, and chemoresistance. Furthermore, L1CAM in CRC not only promotes cell growth and survival, but the L1CAM secreted by tumor cells also makes itself more aggressive 48, thus, promoting the infiltration and metastasis of CRC. Our study suggests that tRF-22/27/32 are highly likely to be involved in the development of CRC by regulating cell proliferation, regulating the cell cycle, and interfering with biological processes such as cell adhesion. Finally, upon investigating the expression pattern of tRF-22/27/32 in the plasma of CRC patients, we found that compared with normal subjects, tRF-22/27/32 were also significantly higher in the plasma of CRC patients. As diagnostic markers for CRC, the AUC values of tRF-22/27/32 were 0.736, 0.699, and 0.786, respectively. These values were higher than those of the conventional markers CEA (0.600) and CA199 (0.562), affirming the potential of tRF-22/27/32 as plasma markers for CRC screening. In addition, the study by Xue et al. shows that the combination of tsRNA-MetCAT-37, tsRNA-ValTAC-41, and CA199 in pancreatic cancer can increase the AUC value of the latter in diagnosing pancreatic cancer 49. In recent years, studies on gene expression regulation by tRFs at different levels have been increasing, and the molecular mechanism of its involvement in the occurrence and development of cancer has been gradually revealed. The establishment and improvement of tRFs databases will provide new directions and capabilities for the future diagnosis and treatment of CRC.

Overall, our research demonstrates that tRF-22/27/32, which are closely related to Glu-Gln metabolism and are highly expressed in CRC. Moreover, these tRFs participate in the occurrence and development of CRC by regulating cell proliferation and cell cycle, and interfering cell adhesion. We propose the use of tRF-22/27/32 as potential tumor treatment targets and plasma markers for CRC screening. However, this study still has some limitations and challenges. Further exploration and experimentation are needed to fully understand the relationship between tRF-22/27/32 and the prognosis of CRC patients.

## Supplementary Material

Supplementary figures and tables.

## Figures and Tables

**Table 1:** Demographic and clinical characteristic of patients with Colorectal cancer in 6 sequenced data sets

Cohort	SRP107326	SRP166942	SRP183064	SRP193100	SRP344867	SRP289772
**Tissues**						
Primary Tumor	104	3	6	2	5	20
Adjacent Normal	104	3	6	2	5	32 (Normal volunteers)
**Age**						
Median	61.86	57.67	-	-	-	63
Range	39-85	49-70	-	-	-	30-83
**Sex**						
Male	59	-	-	-	-	22
Female	45	-	-	-	-	30
**Race**						
Asian	104	3	6	2	5	-
Black						-
White						-
American Indian						-
**Location**						
Colon	66	1	-	0	-	47
Rectum	38	2	-	2	-	5
**Stage**					-	
0	2	0	-	0	-	8
Ⅰ	18	0	-	0	-	1
Ⅱ	29	0	-	0	-	4
Ⅲ	42	3	-	2	-	4
Ⅳ	13	0	-	0	-	3

**Table 2:** Correlation analysis of tRF-22/27/32 with clinical data of patients of plasma origin

Clinical pathological indexes		No. of patients	p-value of tRF-22 (High-Low^1^)	p-value of tRF-27 (High-Low)	p-value of tRF-32 (High-Low)
Age	≥75 <75	8 (20.51%) 31 (79.49%)	0.695	0.235	0.013*
Sex	Male Female	23 (57.50%) 17 (42.50%)	0.749	0.749	0.616
Size	≥5cm <5cm	18 (46.15%) 21 (53.85%)	0.111	0.341	1.000
Tumor location	Colon Rectum	33 (82.50%) 7 (17.50%)	1.000	1.000	0.432
Hemicolon	Left Right	25 (64.10%) 14 (35.90%)	1.000	0.191	0.740
Tumor differention	Well Poor	27 (71.05%) 11 (28.95%)	1.000	1.000	0.147
T stage	T1-T2 T3-T4	7 (19.44%) 29 (80.56%)	1.000	0.408	0.200
Lymph node	Positive Negative	16 (41.03%) 23 (58.97%)	0.333	0.333	0.011*
Metastasis	Positive Negative	4 (10.26%) 35 (89.74%)	0.605	1.000	1.000
TNM stage	I-II III-IV	20 (54.05%) 17 (45.95%)	0.746	0.746	0.104
CEA	High Low	13 (33.33%) 26 (66.67%)	0.096	0.320	0.736
CA199	High Low	12 (30.77%) 27 (69.23%)	0.176	0.501	0.174
Nerve/vascular invasion	Positive Negative	18 (51.43%) 17 (48.57%)	0.315	0.092	0.005**
MMR^2^	dMMR pMMR	3 (7.50%) 37 (92.50%)	1.000	0.231	0.565

1 According to Kolmogorov-Smirnov normality test, tRF-32 obeys the Gaussian distribution, use the arithmetic mean to distinguish its high/low expression. Both tRF-22 and tRF-27 do not obey the Gaussian distribution, use the median to distinguish their high/low expression. 2 MLH1, MSH2, MSH6, and PMS2 were all positive for pMMR (normal expression), and 1 or more negative for dMMR (deletion)

## Abbreviations

AGXT: Alanine-glyoxylate aminotransferase
ASL: Argininosuccinate lyase
BCKDK: Branched chain amino acids α Ketate dehydrogenase kinase
CEA: Carcinoembryonic antigen
CRC: Colorectal cancer
DAVID: Database for Annotation, Visualization, and Integrated Discovery
ESCC: Esophageal squamous cell carcinoma
FOLH1: Folate hydrolase 1
OB: Fecal occult blood test
GO: Gene Ontology
Glu: Glutamate
GLS: Glutaminase
Gln: Glutamine
GLUL: Glutamine ligase
GPS1: G protein pathway inhibitor 1
KEGG: Kyoto Encyclopedia of Genes and Genomes
MMR: Mismatch repair
PR: Precision-recall
PCD: Programmed cell death
qRT-PCR: Quantitative real-time PCR
ROC: Receiver operating characteristic
RIMKLA: Ribosomal modification protein RimK-like family member A
SRA: Sequence Read Archive
sncRNAs: Small non-coding RNAs
tRNA: Transfer RNA
tRFs: tRNA-derived RNA fragments
TAMs: Tumor-associated macrophages
