# Supplementary material for: New plasma diagnostic markers for colorectal cancer: transporter fragments of glutamate tRNA origin
Source: J Cancer. 2024 Jan 12;15(5):1299–313. doi: 10.7150/jca.92102 (PMC10861818; doi:10.7150/jca.92102)
Supplement: Supplementary file 1 — Supplementary figures and tables. [file jcav15p1299s1.pdf]

Supplementary Material

New plasma diagnostic markers for colorectal cancer: transporter fragments of glutamate tRNA origin

Changda Ye 1,2†, Fu Cheng1,2†, Luji Huang1,2†, Kang Wang1,2, Lin Zhong1,2, Qinuo Ju4, Yan Lu3*, Manzhao Ouyang1,2*

Table S1. Primers used for real-time PCR assay

Reverse transcription primers for tRFs	Sequence (5'→3')	
tRF-22-RNLNK88KL	GTCGTATCCAGTGCAGGGTCCGAGGTATTCGCACTGGATACGACTCCCTG	
tRF-27-Z3M8ZLSSXUL	GTCGTATCCAGTGCAGGGTCCGAGGTATTCGCACTGGATACGACTCTCGC	
tRF-32-0668K87SERM4P	GTCGTATCCAGTGCAGGGTCCGAGGTATTCGCACTGGATACGACACACCG	
qRT-PCR primers for tRFs	Forward sequence (5'→3')	Reverse sequence (5'→3')
tRF-22-RNLNK88KL	GGGGTTCGATTCCCGGT	AGTGCAGGGTCCGAGGTATT
tRF-27-Z3M8ZLSSXUL	TGGTCGTGGTTGTAGTCCGT	AGTGCAGGGTCCGAGGTATT
tRF-32-0668K87SERM4P	GCCCGGGTTCGACTCC	AGTGCAGGGTCCGAGGTATT
U6	TGCTTCGGCAGCACATATAC	TCACGAATTTGCGTGTCATC

Table S2. A List of the top 50 tRFs with sequences in the intersection tRFs, sorted by the absolute value of LogFC

Mintbase ID	tRF Sequences (5'~3')	LogFC	Type	P.Value	adj.P.Val	Amino acid
tRF-30-IK9NJ4S2I7L7	ATGGGTGGTTCAGTGGTAGAATTC TCGCCT	3.410	i-tRF	7.47E-39	1.73E-35	Gly
tRF-27-Z3M8ZLSSXUL	TTGGTCGTGGTTGTAGTCCGTGCG AGA	3.240	i-tRF	1.59E-36	9.22E-34	Glu
tRF-22-FSXMSL73O	AGCCGTGATCGTATAGTGGTTA	-2.871	5'-tRF	8.61E-38	9.97E-35	His
tRF-29-JYSWRYVMMVHX	CATTGGTCGTGGTTGTAGTCCGTG CGAGA	2.866	i-tRF	5.50E-29	8.49E-27	Glu
tRF-32-0668K87SERM4P	ACCCAGGCGGCCCGGGTTCGACT CCCGGTGTG	2.708	i-tRF	8.13E-27	7.24E-25	Glu
tRF-21-FSXMSL73E	AGCCGTGATCGTATAGTGGTT	-2.623	5'-tRF	4.65E-36	1.79E-33	His
tRF-28-I8W47W1R7HX	ATTGGTCGTGGTTGTAGTCCGTGC GAGA	2.563	i-tRF	1.48E-27	1.63E-25	Glu
tRF-23-FSXMSL730H	AGCCGTGATCGTATAGTGGTTAG	-2.559	5'-tRF	5.28E-37	4.07E-34	His
tRF-36-DBNIB9I1KQSX0DB	AAGAACTGCTAACTCATGCCCCC ATGTCTAACAACA	2.504	i-tRF	1.23E-15	1.58E-14	Ser
tRF-30-Z3M8ZLSSXUOL	TTGGTCGTGGTTGTAGTCCGTGCG AGAATA	2.473	i-tRF	8.01E-30	1.86E-27	Glu
tRF-16-88481DD	TCCGGCTCGGAGGACC	2.418	3'-tRF	5.63E-17	9.52E-16	Tyr
tRF-33-OR183O9EDJHPD3	GACCCAGTGGCCTAATGGATAAG GCATCAGCCT	-2.410	5'-half	7.24E-27	6.70E-25	Arg
tRF-29-I8W47W1R7HE6	ATTGGTCGTGGTTGTAGTCCGTGC GAGAA	2.386	i-tRF	1.27E-22	5.27E-21	Glu
tRF-28-Z3M8ZLSSXUD6	TTGGTCGTGGTTGTAGTCCGTGCG AGAA	2.370	i-tRF	1.88E-21	6.79E-20	Glu
tRF-16-884U1DD	TCCGGCTCGAAGGACC	2.368	3'-tRF	1.39E-21	5.09E-20	Tyr
tRF-30-JYSWRYVMMV5B	CATTGGTCGTGGTTGTAGTCCGTG CGAGAA	2.344	i-tRF	1.03E-24	6.12E-23	Glu
tRF-24-FSXMSL732Z	AGCCGTGATCGTATAGTGGTTAGT	-2.312	5'-tRF	3.14E-36	1.45E-33	His
tRF-32-10I9BZBZOS4Y1	AGGAGATTTCAACTTAACTTGACC GCTCTGAC	2.306	3'-tRF	4.64E-15	5.29E-14	Val
tRF-41-DBNIB9I1KQSX0DIJE	AAGAACTGCTAACTCATGCCCCC ATGTCTAACAACATGGCT	2.299	i-tRF	9.26E-19	2.26E-17	Ser
tRF-22-EXEY0VWUJ	ACTTAACTTGACCGCTCTGACC	2.295	3'-tRF	9.54E-25	5.81E-23	Val
tRF-22-WKM2736ZQ	TCGGGCGGGAGTGGTGGCTTTT	2.290	tRF-1	1.60E-29	3.19E-27	Lys
tRF-27-M0IBB7Z92K1	CGGAGATGAAAACCTTTTTCCAA GGAC	-2.247	i-tRF	1.10E-32	3.65E-30	Thr
tRF-31-LSM1M3WE8SSP0	CGCCGCGGCCCGGGTTCGATTCCC GGTCAGG	2.212	i-tRF	3.15E-18	6.88E-17	Glu
tRF-21-739P8WQ0B	GTGGTTGTAGTCCGTGCGAGA	2.195	i-tRF	4.37E-26	3.37E-24	Glu
tRF-33-10I9BZBZOS4YV	AGGAGATTTCAACTTAACTTGACC GCTCTGACC	2.155	3'-tRF	5.67E-16	7.76E-15	Val
tRF-29-IK9NJ4S2I71V	ATGGGTGGTTCAGTGGTAGAATTC TCGCC	2.149	i-tRF	1.76E-27	1.85E-25	Gly
tRF-22-WB8US5652	TCGAATCCGAGTCACGGCACCA	-2.133	3'-tRF	2.13E-29	3.61E-27	His
tRF-31-I8W47W1R7HFEB	ATTGGTCGTGGTTGTAGTCCGTGC GAGAATA	2.114	i-tRF	1.91E-18	4.39E-17	Glu
tRF-21-WKM2736ZE	TCGGGCGGGAGTGGTGGCTTT	2.064	tRF-1	2.52E-28	3.44E-26	Lys
tRF-29-9M739P8WQ0EB	TGGTCGTGGTTGTAGTCCGTGCGA GAATA	2.057	i-tRF	5.52E-23	2.45E-21	Glu
tRF-22-739P8WQ0F	GTGGTTGTAGTCCGTGCGAGAA	2.042	i-tRF	1.02E-22	4.30E-21	Glu
tRF-34-D4ZWRNU3KQ9M29	AAGTGTTTGTGGGTTTAAGTCCCA TTGGTCTAGC	2.029	3'-tRF	6.18E-17	1.03E-15	Asn

tRF-22-XSXMSL73O	TGCCGTGATCGTATAGTGGTTA	-2.023	5'-tRF	1.69E-22	6.73E-21	His
tRF-21-XSXMSL73E	TGCCGTGATCGTATAGTGGTT	-2.004	5'-tRF	4.48E-24	2.47E-22	His
tRF-18-8R1546D2	TCCCCAGTACCTCCACCA	2.000	3'-tRF	2.77E-30	7.14E-28	Ala
tRF-18-INVDRID1	ATGTTTAGACGGGCTCAC	-1.998	i-tRF	2.18E-29	3.61E-27	Phe
tRF-33-P4R8YP9LON4VDP	GCATGGGTGGTTCAGTGGTAGAAT TCTCGCCTG	1.995	5'-half	5.83E-31	1.69E-28	Gly
tRF-21-WB8US565D	TCGAATCCGAGTCACGGCACC	-1.976	3'-tRF	2.09E-26	1.79E-24	His
tRF-35-D4ZWRNU3KQ9MV1	AAGTGTTTGTGGGTTTAAGTCCCA TTGGTCTAGCC	1.974	3'-tRF	3.29E-13	2.70E-12	Asn
tRF-35-FDXXE6XRK4WO2F	AGAACTGCTAACTCATGCCCCCAT GTCTAACAACA	1.958	i-tRF	1.74E-16	2.61E-15	Ser
tRF-31-J87383RPD9W1E	CATGGGTGGTTCAGTGGTAGAATT CTCGCCT	1.946	i-tRF	2.31E-23	1.07E-21	Gly
tRF-34-Q99P9P9NH57S15	GCTTCTGTAGTGTAGTGGTTATCA CGTTCGCCTC	1.923	5'-half	2.31E-24	1.30E-22	Val
tRF-26-OB1690PQR3E	GAAAAAGTCATGGAGGCCATGGG GTT	1.922	5'-tRF	1.88E-23	9.08E-22	Ser
tRF-43-7Z8L8NRS9NS334L2DB	GTTTTTCATATCATTGGTCGTGGTT GTAGTCCGTGCGAGAATA	1.915	i-tRF	1.02E-16	1.59E-15	Glu
tRF-36-PW5SVP9N15WV7W0	GCCGTGATCGTATAGTGGTTAGTA CTCTGCGTTGTG	1.908	5'-tRF	4.86E-19	1.25E-17	His
tRF-23-XSXMSL730H	TGCCGTGATCGTATAGTGGTTAG	-1.897	5'-tRF	3.18E-28	4.08E-26	His
tRF-35-P4R8YP9LON4VN1	GCATGGGTGGTTCAGTGGTAGAAT TCTCGCCTGCC	1.891	5'-tRF	1.43E-24	8.25E-23	Gly
tRF-30-V29K9UV36562	TAGGATGGGGTGTGATAGGTGGC ACGGAGA	1.891	5'-tRF	1.84E-23	9.08E-22	Gln
tRF-36-P4R8YP9LON4VN1B	GCATGGGTGGTTCAGTGGTAGAAT TCTCGCCTGCCA	1.889	5'-tRF	2.05E-19	5.44E-18	Gly
tRF-17-YSQSD2J	TTCCGGCTCGAAGGACC	1.882	3'-tRF	6.52E-17	1.07E-15	Tyr

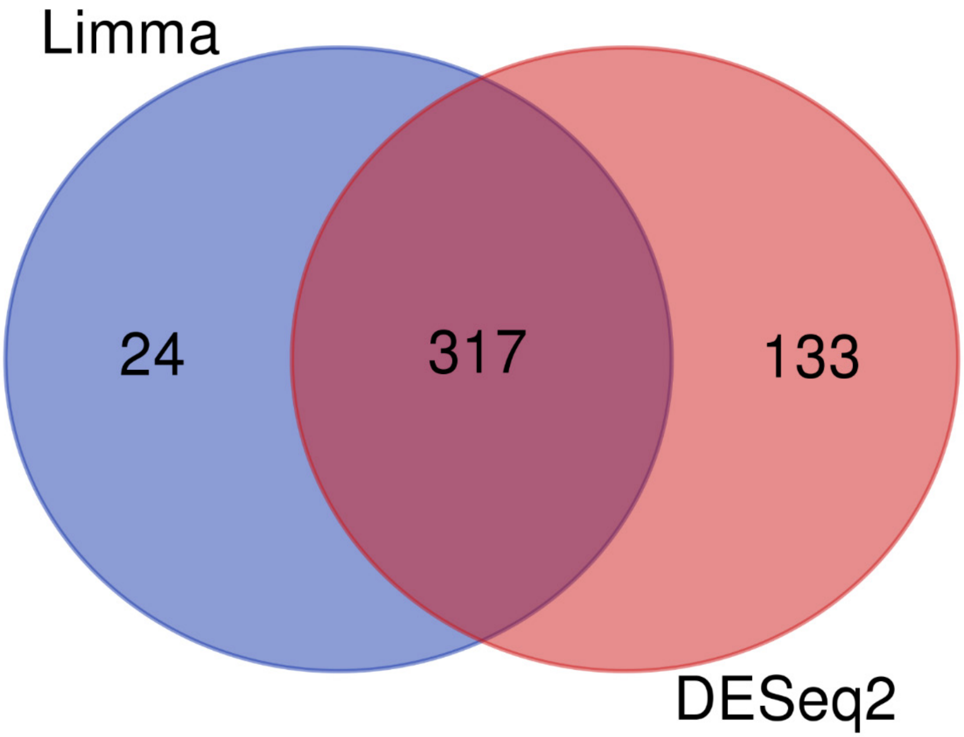

Figure S1. Intersection of variance analysis results of Limma package and DEseq2 package.

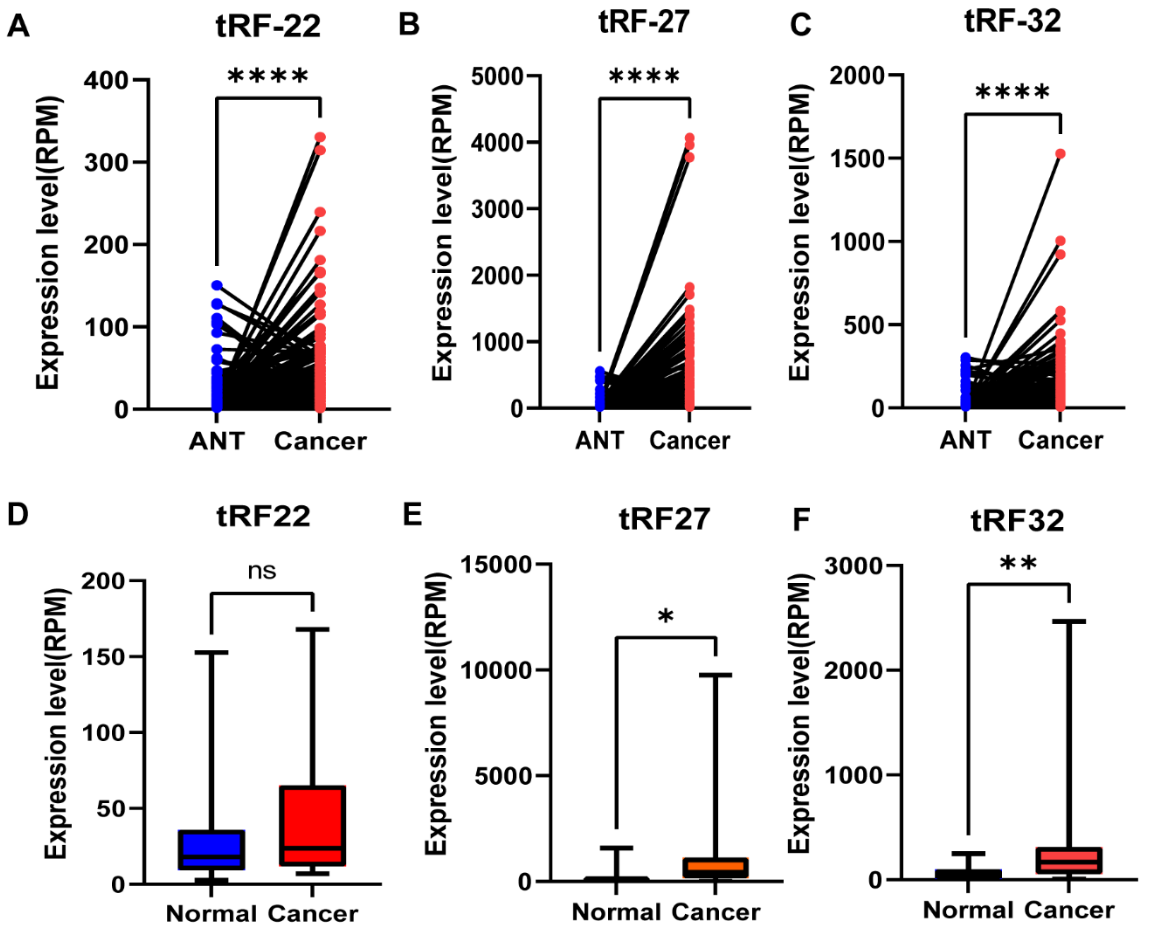

Figure S2. Expression differential analysis of tRF-22/27/32. Differential expression of tRF-22/27/32 in the training set (A~C) and independent validation set (D~F) was analyzed using paired t-test and unpaired t-test.

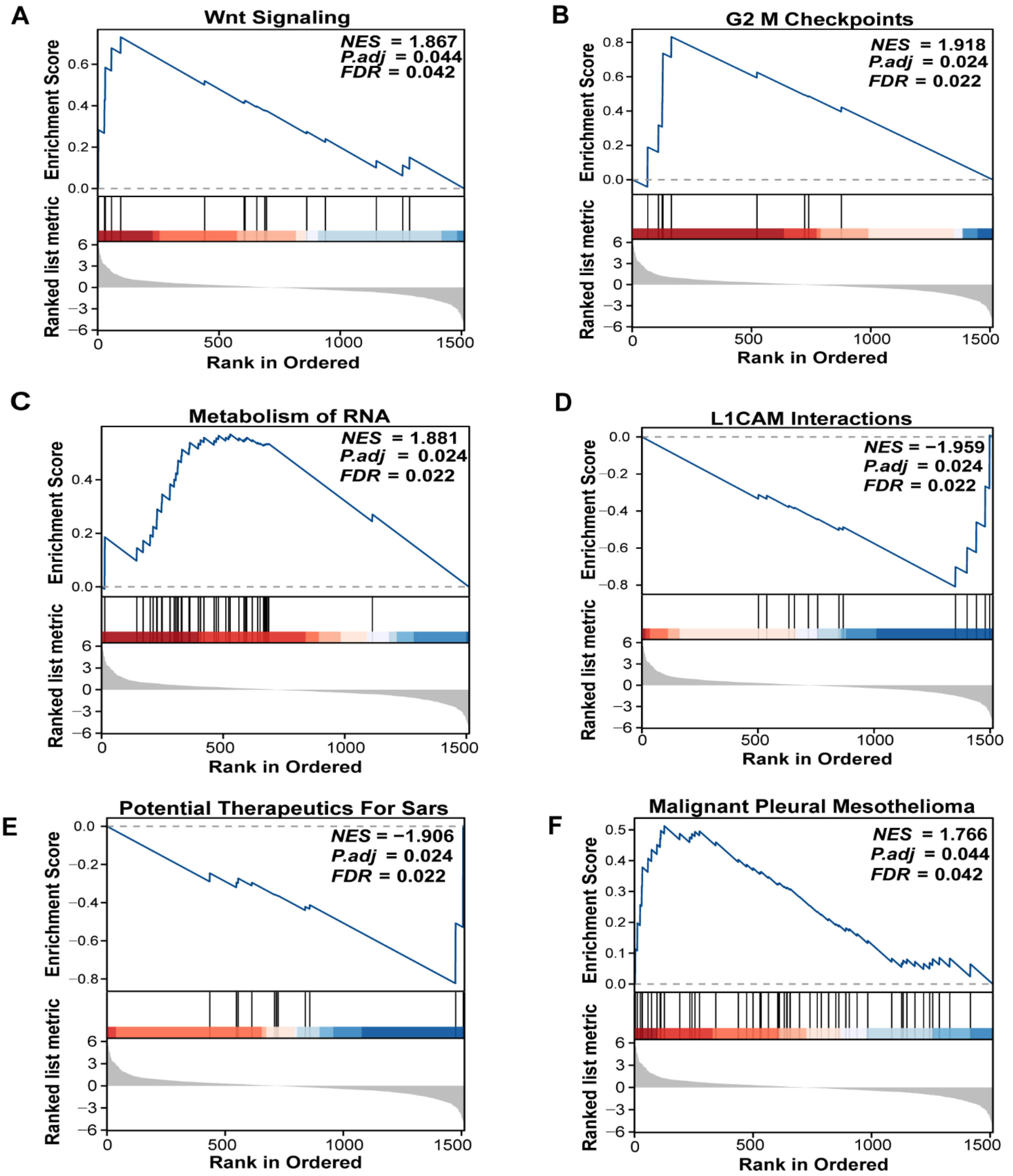

Figure S3. GSEA analysis of target genes. (A~F) CRC target genes RNAseq data obtained from TCGA for GSEA analysis, this figure shows the six pathways with P.adj<0.05.

A

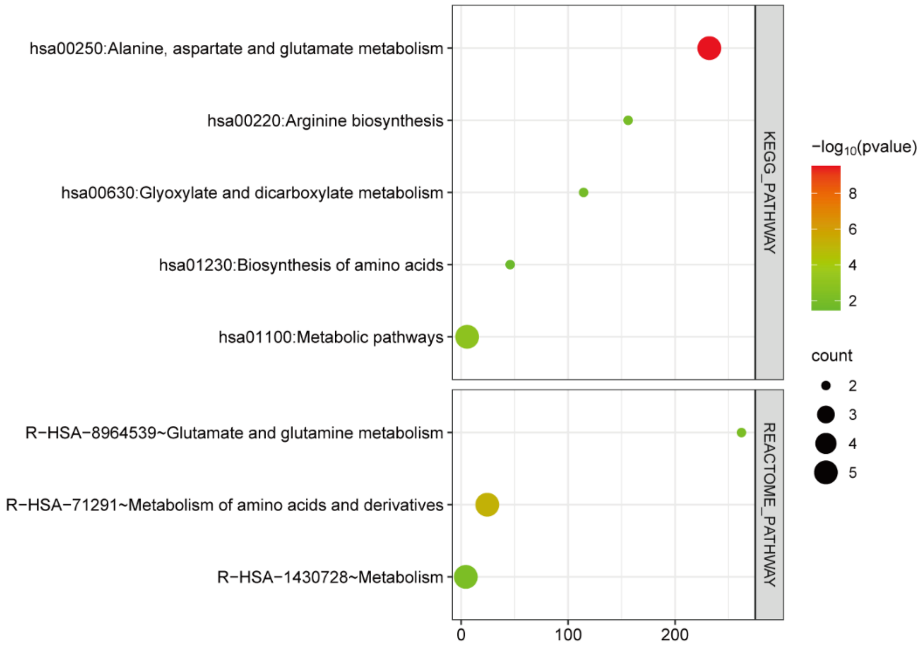

Figure S4. Enrichment analysis of Glu metabolism genes significantly associated with tRF-22/27/32.

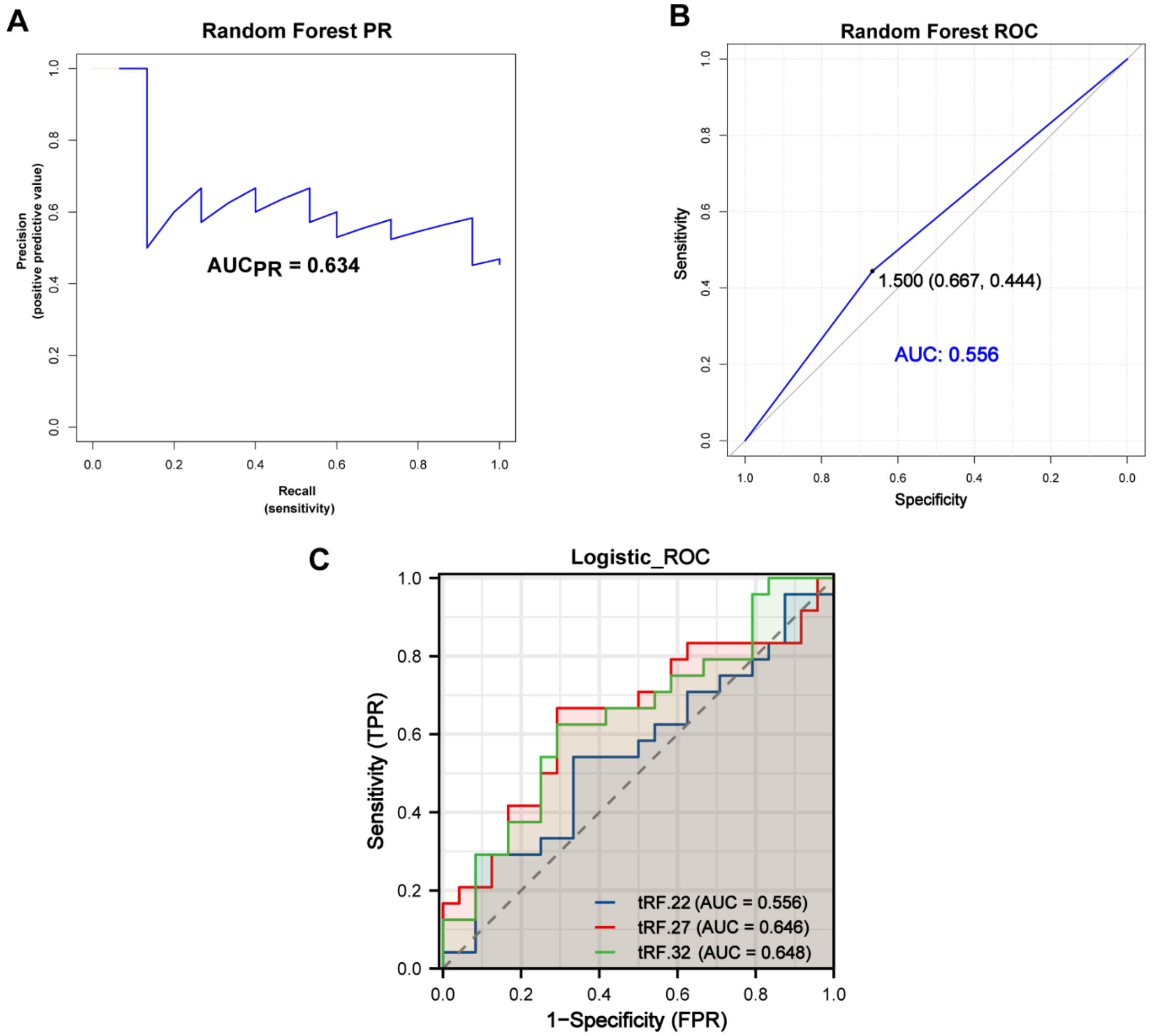

Figure S5. Diagnostic efficacy of qRT-PCR results of tRF-22/27/32 in 24 pairs of CRC cancer and paraneoplastic tissues for CRC under different models. (A~B) Evaluation of the diagnostic efficacy of tRF-22/27/32 qRT-PCR results of 24 pairs of tissues for CRC when random forest model was applied (ROC-AUC=0.556; PR-AUC=0.634); (C) tRF-22/27/32 qRT-PCR results of 24 pairs of tissues when logistic regression model alone was applied diagnostic efficacy (ROC-AUC of 0.556/0.646/0.648 for tRF-22/27/32, respectively).

A

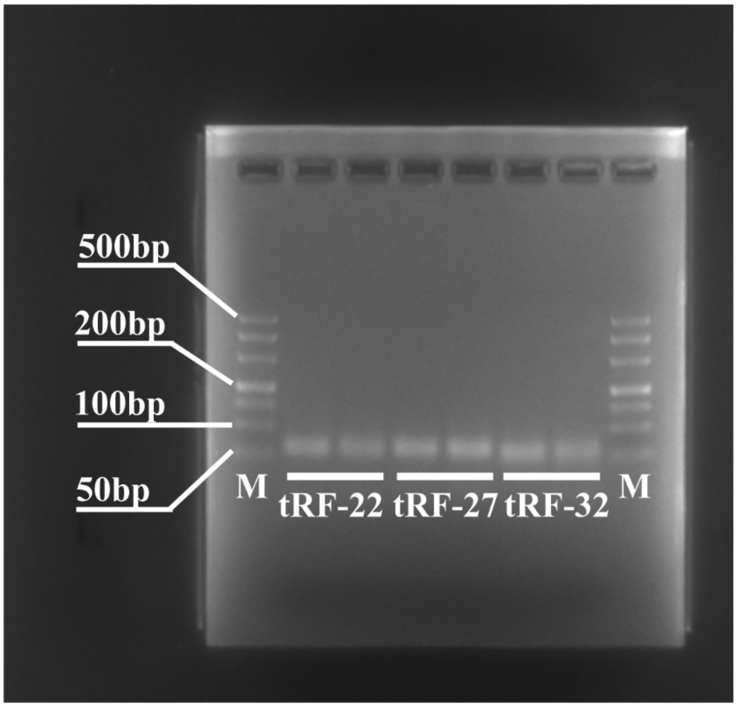

Figure S6. Agarose gel electrophoresis of tRF-22/27/32 qRT-PCR products (two replicates were set for each tRF).

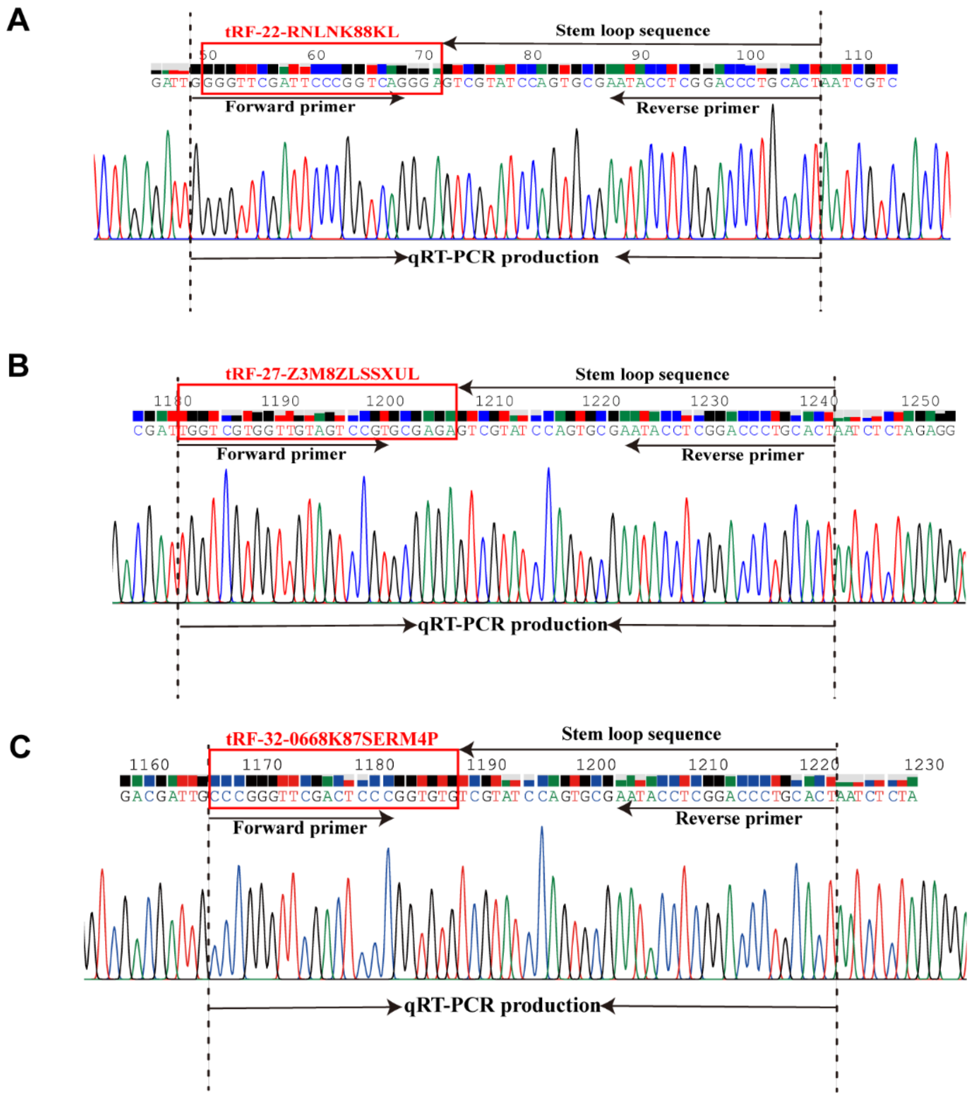

Figure S7. Sanger sequencing experiments were performed to determine qRT-PCR amplification product sequence information. (A~C) Sanger sequencing experiments were performed using tRF-22/27/32 qRT-PCR products to determine that the amplification products were the target products.
